# A Regulatory Network Controls *cabABC* Expression Leading to Biofilm and Rugose Colony Development in *Vibrio vulnificus*

**DOI:** 10.3389/fmicb.2019.03063

**Published:** 2020-01-17

**Authors:** Seung-Ho Hwang, Jin Hwan Park, Byungho Lee, Sang Ho Choi

**Affiliations:** National Research Laboratory of Molecular Microbiology and Toxicology, Center for Food Safety and Toxicology, Department of Agricultural Biotechnology, Seoul National University, Seoul, South Korea

**Keywords:** *Vibrio vulnificus*, biofilm, extracellular matrix protein, colony morphology, transcriptional regulator

## Abstract

Biofilms provide bacteria with protection from environmental stresses and host immune defenses. The pathogenic marine bacterium *Vibrio vulnificus* forms biofilms and colonizes environmental niches such as oysters. The *cabABC* operon encodes an extracellular matrix protein CabA and the corresponding type I secretion system, which are essential for biofilm and rugose colony development of *V. vulnificus*. In this study, molecular biological analyses revealed the roles of three transcriptional regulators BrpR, BrpT, and BrpS in the regulatory pathway for the *cabABC* operon. BrpR induces *brpT* and BrpT in turn activates the *cabABC* operon in a sequential cascade, contributing to development of robust biofilm structures. BrpT also activates *brpS*, but BrpS represses *brpT*, constituting a negative feedback loop that stabilizes *brpT* expression. BrpT and BrpS directly bind to specific sequences upstream of *cabA*, and they constitute a feedforward loop in which BrpT induces *brpS* and together with BrpS activates *cabABC*, leading to precise regulation of *cabABC* expression. Accordingly, BrpS as well as BrpT plays a crucial role in complete development of rugose colonies. This elaborate network of three transcriptional regulators BrpR, BrpT, and BrpS thus tightly controls *cabABC* regulation, and contributes to successful development of robust biofilms and rugose colonies in *V. vulnificus*.

## Introduction

Bacteria exist as free-living planktonic cells or surface-attached complex biofilm communities in the environment (Hall-Stoodley et al., 2004). Biofilms provide bacteria with protection from various stresses such as nutrient limitation and desiccation, as well as antimicrobial agents and host immune defenses during infection (Flemming et al., 2016). Thus, biofilm formation is important for niche colonization and development of persistent bacterial infections (Costerton et al., 1999; Yildiz and Visick, 2009). Biofilm formation involves sequential developmental stages comprised of initial surface attachment, microcolony formation, maturation into three-dimensional biofilms, and dispersal of bacterial cells from mature biofilms (Watnick and Kolter, 2000). Mature biofilms are specialized and highly differentiated communities of bacteria sheathed in an extracellular polymeric matrix (Flemming and Wingender, 2010). The biofilm matrix mainly consists of polysaccharides, proteins, nucleic acids, and lipids; the spatially organized matrix provides beneficial functions for biofilm communities such as resource capture, enzyme retention, and influx inhibition of toxic chemicals (Flemming et al., 2016). Variations in biofilm matrix production can alter the colony morphology, affecting the rugosity and opacity of the apparent colony morphotypes (Yildiz and Visick, 2009; Serra et al., 2013; Park et al., 2015a).

Bis-(3′–5′)-cyclic dimeric guanosine monophosphate (c-di-GMP) is a universal bacterial second messenger and a key player in the molecular decision between the planktonic and biofilm lifestyles (Hengge, 2009). It is synthesized by diguanylate cyclases (DGCs) containing the GGDEF domain and degraded by c-di-GMP-specific phosphodiesterases (PDEs) harboring the EAL or HD-GYP domain (Krasteva et al., 2012). The DGCs and PDEs often carry sensory input domains, and thus control the intracellular c-di-GMP levels precisely in response to various extracellular signals such as light, oxygen, nitric oxide, and nutrient levels (Boyd and O’Toole, 2012; McDougald et al., 2012). Diverse effector components, including regulatory proteins and riboswitches, bind c-di-GMP molecules and regulate downstream target pathways at the transcriptional, post-transcriptional, and post-translational levels (Hengge, 2009; Conner et al., 2017). Generally, increased intracellular c-di-GMP levels lead to stimulation of biofilm formation and repression of motility and virulence (Hengge, 2009).

The pathogenic marine bacterium *Vibrio vulnificus* is a causative agent of food-borne diseases ranging from gastroenteritis to life-threatening septicemia (Oliver, 2015). Raw oysters serve as the primary infection route of *V. vulnificus*, and the pathogen colonizes and persists in oyster populations forming biofilms (Froelich and Oliver, 2013; Park et al., 2016; Pu and Rowe-Magnus, 2018; Pu et al., 2018). Biofilm formation of *V. vulnificus* is induced by elevated levels of intracellular c-di-GMP, which leads to significant changes in gene expression profiles (Nakhamchik et al., 2008; Park et al., 2015b; Chodur and Rowe-Magnus, 2018). Among the genes positively regulated by elevated c-di-GMP levels, the *brp* locus and the *cabABC* operon are essential for c-di-GMP-induced biofilm phenotypes (Guo and Rowe-Magnus, 2010; Park et al., 2015a). The *brp* locus consists of nine genes (*brpABCDFHIJK*), which are responsible for production and secretion of exopolysaccharide (Guo and Rowe-Magnus, 2010). The *cabABC* operon encodes a calcium-binding matrix protein CabA, which is essential for the development of biofilm structure and rugose colony morphology, along with CabB and CabC constituting a type I secretion system for CabA (Park et al., 2015a).

C-di-GMP-dependent biofilm formation by *V. vulnificus* also requires two transcriptional regulators BrpR and BrpT (Guo and Rowe-Magnus, 2010). BrpR and BrpT are homologs of *Vibrio cholerae* VpsR and VpsT, respectively, which are known to bind c-di-GMP directly and regulate transcription of biofilm-associated genes in *V. cholerae* (Krasteva et al., 2010; Zamorano-Sanchez et al., 2015; Hsieh et al., 2018). It was recently reported that the expression of the *brp* locus is positively regulated by BrpT and negatively regulated by BrpS in *V. vulnificus*. BrpS is another VpsT-type regulator whose gene is adjacent to *brpT* (Chodur and Rowe-Magnus, 2018). However, the roles of the three transcriptional regulators BrpR, BrpT, and BrpS in the regulation of *cabABC*, as well as their regulatory hierarchy in *V. vulnificus* have not been well defined. In the present study, we conducted transcript analyses and structural analyses of *V. vulnificus* biofilms and demonstrated that BrpR and BrpT activate the *cabABC* operon in a sequential manner, and thus play an essential role in development of robust biofilm structures. The role of BrpS in the regulatory pathway was also examined, and our results suggested that the expression of *brpT* is regulated by a negative feedback loop involving BrpS. Molecular biological analyses were performed and revealed that both BrpT and BrpS activate *cabABC* by directly binding to specific sequences in the regulatory region, constituting a coherent feedforward loop. Microscopic analyses indicated that BrpS, along with BrpR and BrpT, is required for the complete development of rugose colonies. Together, our results suggest that the three transcriptional regulators BrpR, BrpT, and BrpS collaboratively regulate the *cabABC* operon for robust biofilm and rugose colony development.

## Results

### BrpR and BrpT Positively Regulate *cabABC* as a Sequential Cascade

**FIGURE 1:**
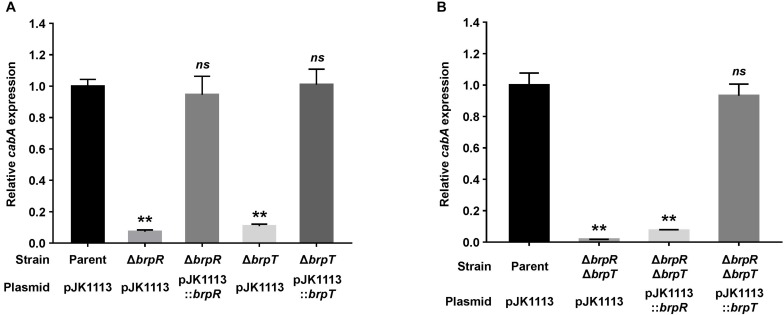
Deletion of *brpR* and *brpT* eliminates *cabA* expression. **(A,B)** Total RNA was isolated from biofilms of the *V. vulnificus* strains grown in microtiter plates. The level of *cabA* transcript was determined by qRT-PCR, and the parent strain was set to 1. Error bars represent the SD from three independent experiments. ^∗∗^, *p* < 0.005 relative to the parent strain; *ns*, not significant. Parent (pJK1113), parent strain; Δ*brpR* (pJK1113), *brpR* mutant; Δ*brpT* (pJK1113), *brpT* mutant; Δ*brpR* (pJK1113::*brpR*) and Δ*brpT* (pJK1113::*brpT*), complemented strains; Δ*brpR*Δ*brpT* (pJK1113), *brpR brpT* double mutant; Δ*brpR*Δ*brpT* (pJK1113::*brpR*) or Δ*brpR*Δ*brpT* (pJK1113::*brpT*), *brpR brpT* double mutant expressing BrpR or BrpT, respectively.

We previously observed that expression of the *cabABC* operon is induced by elevated intracellular c-di-GMP levels (Park et al., 2015b). Since BrpR and BrpT are involved in c-di-GMP-induced biofilm formation (Guo and Rowe-Magnus, 2010), we examined whether the two transcriptional regulators are also involved in the activation of *cabABC*. For this purpose, isogenic mutants lacking *brpR* or *brpT* were generated from *V. vulnificus* JN111, the parent strain previously constructed to manipulate the intracellular c-di-GMP levels (Park et al., 2015a), and the level of *cabA* transcript in biofilm cells of the parent and mutant strains was compared under elevated c-di-GMP levels. The *cabA* expression was almost 10-fold lower in the *brpR* and *brpT* mutants compared with that in the parent strain (Figure 1A). The level of *cabA* expression in the *brpR* and *brpT* mutants was restored to the level in the parent strain by introducing pJK1113::*brpR* and pJK1113::*brpT*, respectively (Figure 1A). The results indicated that both BrpR and BrpT are positive regulators that increase the expression of the *cabABC* operon in either an additive or a sequential manner.

To characterize the functional relationship of BrpR and BrpT in *cabABC* regulation, the *brpR brpT* double mutant was also constructed and the *cabA* expression was determined. In the absence of both *brpR* and *brpT*, there was a significant reduction of *cabA* expression (Figure 1B), but the level of residual *cabA* transcript was comparable with that in the *brpR* or *brpT* single mutant (Figure 1A). This result indicated that the positive regulation of BrpR and BrpT for *cabA* is not additive. When the *brpR brpT* double mutant was complemented with pJK1113::*brpR*, *cabA* expression was not activated in the absence of *brpT* (Figure 1B). However, complementation of the double mutant with pJK1113::*brpT* restored the level of *cabA* expression to the level in the parent strain in the absence of *brpR* (Figure 1B). These results suggested that BrpT activates *cabA* directly, and BrpR activates *cabA* through activation of *brpT* in a sequential manner. Consistent with the suggestion, the expression of *brpT* was greatly reduced in the *brpR* mutant compared with that in the parent strain (Supplementary Figure S1A), whereas *brpR* expression was not affected by the absence of *brpT* (Supplementary Figure S1B). Together, these results suggested that BrpR and BrpT positively regulate *cabABC* expression in a regulatory cascade, in which BrpR activates *brpT*, and BrpT in turn activates *cabABC*.

### BrpR and BrpT Contribute to Robust Biofilm Structures Through Activation of *cabABC*

**FIGURE 2:**
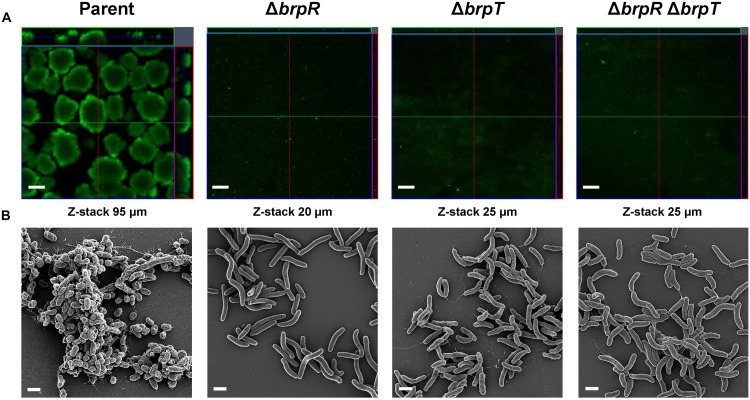
Deletion of *brpR* and *brpT* disrupts biofilm structures. Biofilms of the *V. vulnificus* strains were grown in flow cell chambers under constant flow. **(A)** Biofilms were stained by LIVE/DEAD BacLight Viability Kit (Invitrogen), and CLSM (LSM710, Zeiss) images were acquired at 100 × magnification. The depth of the Z-stack is indicated below the images in μm. **(B)** Biofilms of the strains were fixed, dehydrated, coated with platinum, and visualized using SEM (Supra 55VP, Zeiss) at 10000 × magnification. Scale bars, 100 μm **(A)** and 1 μm **(B)**; Parent, parent strain; Δ*brpR*, *brpR* mutant; Δ*brpT*, *brpT* mutant; Δ*brpR*Δ*brpT*, *brpR brpT* double mutant.

To assess the importance of BrpR and BrpT in biofilm development, biofilm structures of the *V. vulnificus* strains developed in response to elevated c-di-GMP levels were examined in flow cells. As shown in Figure 2A, confocal laser scanning microscopy (CLSM) images demonstrated that the parent strain produced biofilms with robust, large, uniform, and mushroom-like structures. However, the mutant strains lacking *brpR*, *brpT*, or both *brpR* and *brpT* produced weak, sparse, inconsistent, and markedly unstructured biofilms, which were similar to those previously observed when CabA was absent from the biofilm matrix (Park et al., 2015a). Z-stack measurements showed that the depth of the parent strain biofilms was 95 μm, whereas those of the mutant strain biofilms were between 20 and 25 μm. These results indicated that BrpR and BrpT are important for development of robust biofilms.

To examine the effects of BrpR and BrpT on the biofilm structure in more detail, the biofilms developed in flow cells were further examined using scanning electron microscopy (SEM) (Figure 2B). The parent strain produced compact and thick biofilms in which bacterial cells were bound together and encased within an extracellular matrix. Filamentous materials that connected bacterial cells were visible in the biofilm matrix of the parent strain. In contrast, the mutant strains lacking *brpR*, *brpT*, or both *brpR* and *brpT* exhibited only a few bacterial cell clusters that were void of any extracellular matrix surrounding the cells. Since CabA is an essential component of the extracellular matrix (Park et al., 2015a), this observation indicated that *cabABC* expression activated by BrpR and BrpT is crucial for development of the structured matrix. Therefore, these results, combined with the results from transcript analyses (Figure 1), suggested that BrpR and BrpT are essential for activation of the *cabABC* operon, thereby contributing to the structural integrity of *V. vulnificus* biofilms.

### BrpT and BrpS Constitute a Negative Feedback Loop

**FIGURE 3:**
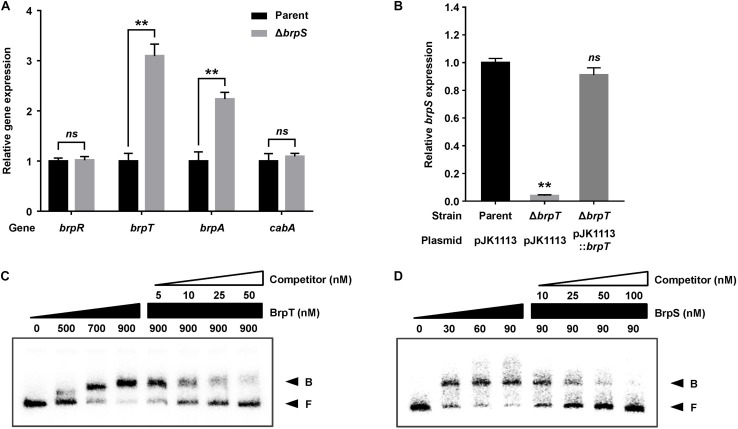
BrpT and BrpS constitute a negative feedback loop. **(A,B)** Total RNA was isolated from biofilms of the *V. vulnificus* strains grown in microtiter plates. The levels of *brpR*, *brpT*, *brpA*, and *cabA* transcripts **(A)** or *brpS* transcript **(B)** were determined by qRT-PCR, and the parent strain was set to 1. Error bars represent the SD from three independent experiments. ^∗∗^, *p* < 0.005 relative to the parent strain; *ns*, not significant. Parent and Parent (pJK1113), parent strain; Δ*brpS*, *brpS* mutant; Δ*brpT* (pJK1113), *brpT* mutant; Δ*brpT* (pJK1113::*brpT*), complemented strain. **(C)** A 358-bp DNA fragment of the *brpS* upstream region (2.5 nM) was radioactively labeled and incubated with increasing amounts of BrpT as indicated in the presence of c-di-GMP (50 μM). For competition analysis, the same but unlabeled 358-bp DNA fragment was used as self-competitor DNA. Various amounts of self-competitor DNA were added as indicated to a reaction mixture containing the probe DNA before the addition of BrpT. **(D)** A 388-bp DNA fragment of the *brpT* upstream region (2.5 nM) was radioactively labeled and incubated with increasing amounts of BrpS as indicated in the presence of c-di-GMP (50 μM). For competition analysis, the same but unlabeled 388-bp DNA fragment was used in the same manner as described above, except that BrpS was added. B, bound DNA; F, free DNA.

To examine whether BrpS is also involved in the pathway for *cabABC* regulation, the isogenic *brpS* mutant was constructed and expression of *brpR*, *brpT*, *brpA*, and *cabA* was compared to the parent strain. The *brpS* deletion did not significantly affect *brpR* expression but increased the level of *brpT* expression about 3-fold (Figure 3A), suggesting that BrpS represses *brpT*. In contrast, the expression of *brpS* was substantially reduced in the absence of *brpT* (Figure 3B), indicating that BrpT activates *brpS*. Electrophoretic mobility shift assays (EMSAs) also revealed that BrpT and BrpS could directly bind to the upstream region of *brpS* and *brpT*, respectively (Figures 3C,D). These results suggested that BrpT activates *brpS*, but BrpS represses *brpT*, forming a negative feedback loop. Since BrpT activates both the *cabABC* operon (Figure 1), as well as the *brp* locus (Chodur and Rowe-Magnus, 2018), both of these loci might be activated by the higher levels of BrpT in the *brpS* mutant. However, only *brpA* expression increased in the *brpS* mutant, and not *cabA* (Figure 3A). This result indicated that additional regulatory factor(s) other than BrpT, which counterbalances the effect of increased BrpT, likely participates in the precise regulation of the *cabABC* operon.

### Determination and Deletion Analysis of the *cabA* Promoter

**FIGURE 4:**
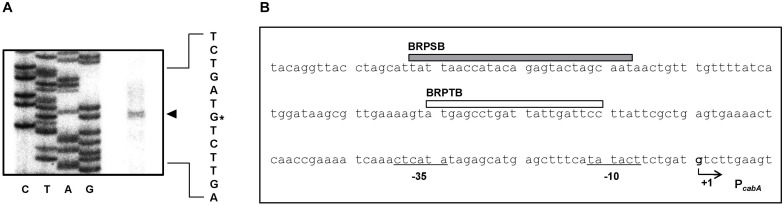
Transcription start site and sequence of the P*_*cabA*_* regulatory region. **(A)** The transcription start site of P*_*cabA*_* was determined by primer extension of the RNA isolated from biofilms of the parent strain. Lanes G, A, T, and C represent the nucleotide sequencing ladders. The asterisk indicates the transcription start site of P*_*cabA*_*. **(B)** The transcription start site of P*_*cabA*_* is indicated by a bent arrow, and the position of the putative –10 and –35 regions are underlined. The sequences for binding of BrpS (BRPSB; gray box) and BrpT (BRPTB; white box) were determined later in this study (Figures 6C,D).

To map the promoter of the *cabABC* operon, the transcription start site was determined by primer extension analysis. A single reverse transcript was produced from primer extension of the RNA isolated from biofilms of the parent strain grown under elevated intracellular c-di-GMP levels (Figure 4A). The 5′-end of the *cabABC* transcript was located 79 bp upstream of the translation initiation codon of *cabA* and was subsequently designated +1 (Figure 4B). The putative promoter constituting the transcription start site was named P*_*cabA*_* to represent the *cabA* promoter, and the sequences for −10 and −35 regions of P*_*cabA*_* were assigned on the basis of similarity to the consensus sequences of *Escherichia coli* σ^70^ promoters (Figure 4B).

**FIGURE 5:**
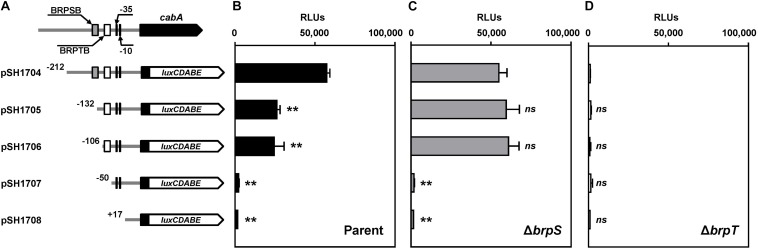
Deletion analysis of the P*_*cabA*_* regulatory region. **(A)** PCR fragments carrying the P*_*cabA*_* regulatory region with 5′-end deletions were subcloned into pBBR-lux to create each pSH reporter. The wild-type P*_*cabA*_* regulatory region is shown on top with the proposed –10 and –35 regions, and the binding sites for BrpS (BRPSB; gray box) and BrpT (BRPTB; white box) were determined later in this study (Figures 6C,D). Solid lines, the upstream region of *cabA*; black blocks, the *cabA* coding region; white blocks, *luxCDABE*. **(B–D)** Cellular luminescence was determined from the parent strain (B, black bars), *brpS* mutant (C, gray bars), and *brpT* mutant (D, white bars) containing each pSH reporter as indicated. Cultures grown to an *A*_600_ of 2.0 were used to measure the cellular luminescence. Error bars represent the SD from three independent experiments. ^∗∗^, *p* < 0.005 relative to the strain containing pSH1704; *ns*, not significant. RLUs, relative light units. Parent, parent strain; Δ*brpS*, *brpS* mutant; Δ*brpT*, *brpT* mutant.

To delineate the *cis*-DNA sequences required for the P*_*cabA*_* activity, pSH reporters carrying varied P*_*cabA*_* regulatory regions, which were deleted up to different 5′-ends and fused transcriptionally to *luxCDABE*, were constructed (Figure 5A). The luminescence produced by pSH1704 carrying P*_*cabA*_* deleted up to −212 was comparable between the parent strain and *brpS* mutant (Figures 5B,C). This was consistent with the comparable levels of *cabA* expression in these two strains (Figure 3A). Compared with pSH1704, the reporters pSH1705 and pSH1706, carrying P*_*cabA*_* deleted up to −132 and −106, respectively, produced significantly reduced luminescence in the parent strain (Figure 5B). However, the relative light units (RLUs) of the *brpS* mutants containing pSH1704, pSH1705, or pSH1706 did not significantly differ (Figure 5C), indicating that the *cis*-DNA sequence required for BrpS-dependent activation of P*_*cabA*_* is deleted in pSH1705 and pSH1706. The reporters pSH1707 and pSH1708 produced the basal level of RLUs in the parent strain (Figure 5B), and the level was comparable with the level produced by any pSH reporter in the *brpT* mutant (Figure 5D). This observation indicated that BrpT-dependent activation is crucial for the P*_*cabA*_* activity and is probably impaired by deletion of P*_*cabA*_* regulatory region up to −50. The results implied that the *cis*-DNA sequences necessary for BrpS and BrpT to activate P*_*cabA*_* encompass the −132 and −50 regions, respectively.

### BrpT and BrpS Directly Bind to P*_*cabA*_*

**FIGURE 6:**
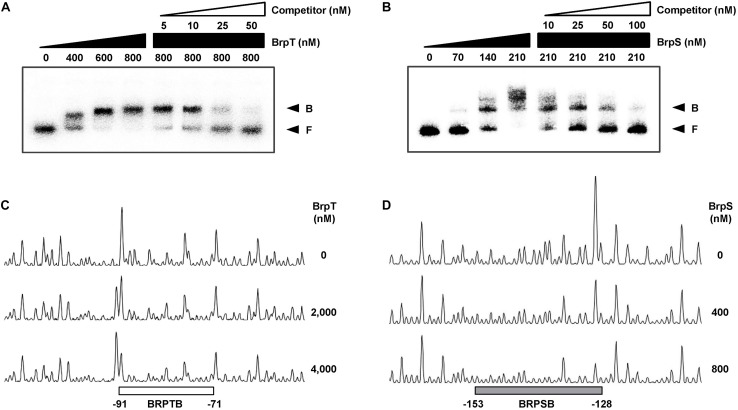
Specific binding of BrpT and BrpS to P*_*cabA*_*. **(A,B)** A 337-bp DNA fragment of the P*_*cabA*_* regulatory region (2.5 nM) was radioactively labeled and incubated with increasing amounts of BrpT **(A)** or BrpS **(B)** as indicated in the presence of c-di-GMP (50 μM). For competition analysis, the same but unlabeled 337-bp DNA fragment was used as a self-competitor DNA. Various amounts of self-competitor DNA were added as indicated to a reaction mixture containing the probe DNA before the addition of BrpT **(A)** or BrpS **(B)**. B, bound DNA; F, free DNA. **(C,D)** A 337-bp DNA fragment of same P*_*cabA*_* regulatory region as above was labeled with 6-FAM and then used as a probe DNA. The 6-FAM-labeled probe DNA (48 nM) was incubated with increasing amounts of BrpT **(C)** or BrpS **(D)** as indicated in the presence of c-di-GMP (50 μM), and then digested with DNase I. The regions protected from DNase I cleavage by BrpT or BrpS are indicated by a white (**C**, BRPTB) or gray box (**D**, BRPSB), respectively. Nucleotide numbers shown are relative to the transcription start site of P*_*cabA*_*.

To examine whether BrpT and BrpS directly bind to the P*_*cabA*_* promoter, EMSAs were performed as shown in Figures 6A,B. For this purpose, the 337-bp labeled DNA probe encompassing the P*_*cabA*_* regulatory region (from −230 to +107) was incubated with increasing amounts of BrpT or BrpS and then subjected to electrophoresis. The addition of BrpT or BrpS to the DNA probe resulted in a retarded band in a concentration-dependent manner (Figures 6A,B). The binding of BrpT and BrpS was specific, because the assays were performed in the presence of poly(dI-dC) as a non-specific competitor. In addition, the same unlabeled 337-bp DNA fragment was used as a self-competitor to confirm the specific binding of BrpT and BrpS. The unlabeled DNA competed for binding of BrpT and BrpS in a dose-dependent manner (Figures 6A,B), confirming that BrpT and BrpS bind specifically to P*_*cabA*_*.

DNase I protection assays were performed using the 337-bp DNA probe encompassing the same region as above to identify the binding sequences for BrpT and BrpS in the P*_*cabA*_* regulatory region. Upon addition of BrpT, the region extending from −91 to −71 (BRPTB, centered at −81) was protected from DNase I digestion (Figure 6C). The addition of BrpS resulted in protection of the region extending from −153 to −128 (BRPSB, centered at −140.5), as shown in Figure 6D. Taken together, these results suggested that BrpT and BrpS directly regulate *cabABC* by binding to the specific sequences of P*_*cabA*_*.

### BrpS Is Important for Complete Development of Rugose Colonies

**FIGURE 7:**
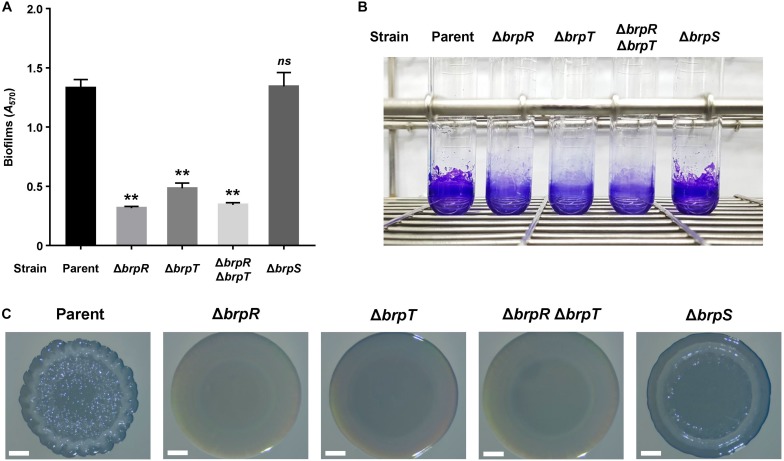
Effects of *brpR*, *brpT*, and *brpS* deletions on biofilm formation and colony morphology. **(A)** Biofilms of the *V. vulnificus* strains were grown in microtiter plates for 24 h, and then stained with 1% crystal violet. The crystal violet was eluted, and its absorbance at 570 nm (*A*_570_) was determined to quantify the biofilms. Error bars represent the SD from three independent experiments. ^∗∗^, *p* < 0.005 relative to the parent strain; *ns*, not significant. **(B)** Biofilms were grown and stained as described above, except that round-bottom test tubes were used. The stained biofilms were photographed using a digital camera (PowerShot G7X Mark II, Canon). **(C)** *V. vulnificus* strains were spotted onto VFMG agar plates and incubated for 36 h. Each colony that represented the mean rugosity from at least three independent experiments was visualized using a stereomicroscope (Stemi 305, Zeiss) at 8 × magnification. Scale bars, 1 mm. Parent, parent strain; Δ*brpR*, *brpR* mutant; Δ*brpT*, *brpT* mutant; Δ*brpR*Δ*brpT*, *brpR brpT* double mutant; Δ*brpS*, *brpS* mutant.

We previously demonstrated that CabA is essential for development of both biofilms and rugose colonies under elevated c-di-GMP levels (Park et al., 2015a). Thus, the role of BrpS as well as BrpR and BrpT on the biofilm- and rugose colony-forming abilities of the *V. vulnificus* strains was examined. Biofilms were grown in microtiter plates and quantified using crystal violet staining assays (Figure 7A). Compared to the parent strain, the level of biofilm formation was significantly reduced in the mutant strains lacking *brpR*, *brpT*, or both *brpR* and *brpT*. This was consistent with the severe reduction of *cabA* expression observed (Figure 1). Meanwhile, biofilm formation of the *brpS* mutant was comparable with that of the parent strain. This could be attributed to the comparable level of *cabA* expression in these two strains (Figure 3A). Biofilm formation of the strains, in the same condition used for Figure 7A but in a larger scale in test tubes, were also similar with those observed in microtiter plates (Figure 7B). This correlation of biofilm formation with the *cabA* expression supported our previous observation that expression of *cabABC* plays a decisive role in biofilm development of *V. vulnificus*.

To further examine the effects of the regulatory proteins on colony rugosity, the parent and mutant strains were grown on agar plates and their colony morphologies were visualized (Figure 7C). The parent strain displayed the rugose colony morphology, but the mutant strains lacking *brpR*, *brpT*, or both *brpR* and *brpT* exhibited the smooth colony morphology. Again, this was expected, based on the reduced *cabA* expression in these mutants (Figure 1). Interestingly, the *brpS* mutant displayed a colony morphology that was distinct from the parent and other mutant strains. The colony of the *brpS* mutant was characterized by a concentric ridge around the rim, but it was less wrinkled than the rugose colony of the parent strain. It has also been previously reported that BrpS is required for the colony rugosity (Chodur and Rowe-Magnus, 2018). This could result from the imbalance between matrix components in the *brpS* mutant. *V. vulnificus* requires production of both the matrix protein CabA and brp-exopolysaccharide to develop rugose colony morphology (Park et al., 2015a). In contrast to the expression of the *cabABC* operon, that of the *brp* locus greatly increased in the *brpS* mutant (Figure 3), leading to overproduction of exopolysaccharides in the mutant strain (Chodur and Rowe-Magnus, 2018). Still, the *brpS* mutant did not show an increase in the level of biofilm formation (Figures 7A,B), indicating that the overproduced exopolysaccharides did not further increase the biofilm biomass. However, this imbalance could lead to significantly altered colony morphology, because proper balance between the matrix components is critical for development of a specific morphology (Serra et al., 2013). These results suggested that BrpS as well as BrpR and BrpT plays an important role in the complete development of rugose colonies as well as elaborate regulation of the *cabABC* operon.

## Discussion

*Vibrio vulnificus* CabA is a structural protein of the extracellular matrix that plays a crucial role in development of robust biofilms and rugose colonies (Park et al., 2015a). Another component in the biofilm matrix of *V. vulnificus* is the exopolysaccharide encoded by the *brp* locus (Guo and Rowe-Magnus, 2010). CabA and brp-exopolysaccharide are functionally associated, and CabA requires brp-exopolysaccharides to develop a robust matrix structure (Park et al., 2015a). It was previously reported that BrpT activates the expression of the *brp* locus (Chodur and Rowe-Magnus, 2018). In the present study, we demonstrated that BrpR and BrpT activate the expression of the *cabABC* operon in a sequential cascade (Figure 1 and Supplementary Figure S1). These observations propose that expression of the *brp* locus and the *cabABC* operon are induced together through BrpR and BrpT under elevated c-di-GMP levels. Consistent with this proposition, the mutant strains deficient in *brpR* or *brpT* formed bare bacterial cells without any extracellular matrix in flow cells (Figure 2), indicating that production of both matrix protein CabA and brp-exopolysaccharide was suppressed. The combined results suggest that the *cabABC* operon and the *brp* locus are coordinately regulated for efficient and successful development of biofilms.

In the present study, we also revealed the regulatory relationship between BrpT and BrpS. A previous study reported that BrpT represses *brpS* in *V. vulnificus* strain ATCC 27562 (Chodur and Rowe-Magnus, 2018). However, in this study, we determined that BrpT and BrpS constitute a negative feedback loop, in which BrpT activates *brpS*, but *brpT* is repressed by BrpS (Figure 3). This discrepancy was not due to strain differences, as reexamination of the regulatory relationship between BrpT and BrpS in the ATCC 27562 strain also identified the same negative feedback loop (Supplementary Figure S2). Another possible explanation is that the transcriptional reporter system they used may not contain the whole *cis*-element sequences required for P*_*brpS*_* activation, since the P*_*brpS*_* reporter exhibited unusually low level of expression compared to other reporters used in the study (Chodur and Rowe-Magnus, 2018). They also reported that *brpS* expression is activated under elevated intracellular c-di-GMP levels, and it makes sense if BrpT, which is induced by elevated c-di-GMP levels (Chodur and Rowe-Magnus, 2018), activates *brpS*, as we proposed.

It is logical to expect that increasing *brpT* expression in the *brpS* mutant would increase expression of both the *cabABC* operon and the *brp* locus. However, although expression of the *brp* locus increased, that of *cabABC* did not in the *brpS* mutant (Figure 3). This result indicates that the effect of BrpT-mediated activation of *cabABC* is counterbalanced by another effect(s) resulting from the *brpS* deletion. One possible explanation is that BrpS also activates the *cabABC* operon. Indeed, BrpS as well as BrpT directly regulates the *cabABC* operon by binding to specific, but distinct sequences in the P*_*cabA*_* regulatory region (Figures 4–6). The positioning of BrpT- and BrpS-binding sequences suggested that both BrpT and BrpS may act as class I activators interacting with the C-terminal domain of RNA polymerase α subunits (Browning and Busby, 2016). These results indicate that BrpT and BrpS activate *cabABC* in a feedforward loop for additive and precise regulation (Alon, 2007). We have also tried to examine the simultaneous binding of BrpT and BrpS to the P*_*cabA*_* regulatory region *in vitro*. However, the specific requirements of buffer composition for the binding of BrpT or BrpS were so different from each other that we could not find any optimized composition for the simultaneous binding of both proteins (see Experimental Procedures). The possible interaction between BrpT and BrpS in activation of P*_*cabA*_* remains to be elucidated.

Although we proposed that BrpS activates the *cabABC* operon, the *cabA* expression (Figure 3A), as well as the P*_*cabA*_* activity (Figures 5B,C), was not reduced in the *brpS* mutant compared with that in the parent strain. This observation could result from the increased *brpT* expression in the absence of BrpS (Figure 3A), which may compensate for the loss of BrpS-dependent activation of *cabA*. In this case, BrpS might not be required for full activation of *cabABC*. However, BrpS was required for complete development of rugose colonies (Figure 7C), possibly by balancing matrix components. Moreover, the region encompassing the BrpS-binding sequence was required for full activation of *cabA* in the parent strain (Figure 5B). This effect was BrpS-dependent, because deletion of the region did not affect *cabA* activation in the absence of BrpS (Figure 5C). These results suggest that *V. vulnificus* requires BrpS for rugose colony development and, at the same time, BrpS contributes to proper *cabABC* expression by activating *cabABC* while repressing *brpT*.

Transcriptional regulators which are homologous to BrpR, BrpT, and BrpS have also been studied in *V. cholerae* and *Vibrio parahaemolyticus*. Best known are VpsR and VpsT, two major regulators of biofilm formation in *V. cholerae*. VpsR and VpsT regulate expression of each other, and both can directly regulate downstream exopolysaccharide and matrix protein genes (Beyhan et al., 2007; Zamorano-Sanchez et al., 2015). On the contrary, BrpR and BrpT act in a sequential manner, and BrpT, not BrpR, directly regulates the *brp* locus and *cabABC* operon. Three regulatory proteins CpsR, CpsQ, and CpsS, corresponding to BrpR, BrpT, and BrpS respectively, also constitute a regulatory pathway for biofilm formation in *V. parahaemolyticus*. These three act in a cascade; While CpsS represses *cpsR*, CpsR activates *cpsQ*, and CpsQ activates expression of downstream genes (Ferreira et al., 2012). In contrast to the role of CpsS, which acts as a master repressor of the pathway, BrpS plays two distinct roles. First, BrpS represses *brpT* in a negative feedback loop. It can stabilize the steady state expression of *brpT* (Dublanche et al., 2006; Chalancon et al., 2012). Second, BrpS, along with BrpT, activates *cabABC*. It makes a difference between the regulation of the *cabABC* operon and the *brp* locus, so the balance in expression of the two loci can be properly adjusted through BrpS. Thus, *V. vulnificus* has a distinct regulatory network, which may be reflected in the differences of biofilm life style and niche occupation between *V. vulnificus* and other *Vibrio* species.

**FIGURE 8:**
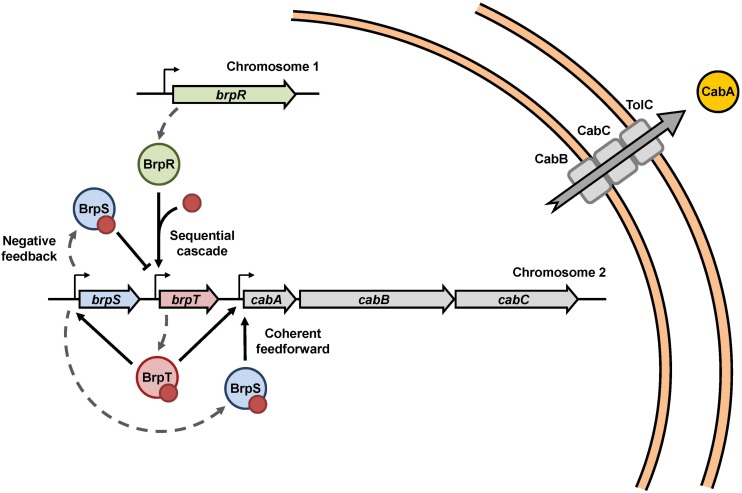
Proposed model for the *cabABC* regulatory network. A regulatory network comprised of the three transcriptional regulators BrpR, BrpT, and BrpS controls expression of the *cabABC* operon. Intracellular c-di-GMP levels can be altered in response to various environmental signals. Under elevated intracellular c-di-GMP levels, BrpR activates *brpT* and *cabABC* in a sequential cascade. BrpT and BrpS constitute a negative feedback loop for *brpT* regulation, where BrpT activates *brpS*, but BrpS represses *brpT*. BrpT and BrpS also form a feedforward loop for positive regulation of *cabABC*. CabA is secreted through a type I secretion system comprised of CabB, CabC, and the outer membrane protein TolC. CabA in the extracellular matrix contributes to development of robust biofilm structures and rugose colony morphology in cooperation with brp-exopolysaccharide. The red dots represent c-di-GMP molecules.

BrpR, BrpT, and BrpS combine to regulate the *cabABC* operon in *V. vulnificus*, and constitute a complex regulatory network as depicted in Figure 8. In summary, under elevated intracellular c-di-GMP levels, BrpR activates *brpT*, and BrpT in turn activates *brp*S and the *cabABC* operon. BrpT and BrpS together constitute a negative feedback loop and a coherent feedforward loop to regulate *brpT* and the *cabABC* operon, respectively. BrpT and BrpS activate *cabABC* expression by directly binding to the specific sequences in the P*_*cabA*_* regulatory region, leading to development of a structured biofilm matrix and rugose colony morphology. Consequently, the combination of three transcriptional regulators BrpR, BrpT, and BrpS elaborately controls *cabABC* expression, driving robust biofilm and rugose colony development by *V. vulnificus*.

## Materials and Methods

### Strains, Plasmids, and Culture Conditions

The strains and plasmids used in this study are listed in Supplementary Table S1. Unless otherwise noted, the *V. vulnificus* strains were grown aerobically in LB medium supplemented with 2.0% (w/v) NaCl (LBS) at 30°C. The *Vibrio fischeri* minimal medium (Cao et al., 2012) containing glycerol (50 mM Tris–HCl, pH 7.2, 50 mM MgSO_4_, 300 mM NaCl, 10 mM KCl, 0.33 mM K_2_HPO_4_, 18.5 mM NH_4_Cl, 10 mM CaCl_2_, and 32.6 mM glycerol) (VFMG) was used for biofilm formation. When required, antibiotics were added to the media at the following concentrations: kanamycin, 100 μg/ml; chloramphenicol, 3 μg/ml. To manipulate intracellular c-di-GMP levels, *V. vulnificus* JN111, which carries *dcpA* encoding a diguanylate cyclase (Nakhamchik et al., 2008) on the chromosome under the control of arabinose-inducible promoter P_BAD_ (Guzman et al., 1995), was constructed previously (Park et al., 2015a). JN111 was used as a parent strain in this study (Supplementary Table S1), and intracellular c-di-GMP levels of the *V. vulnificus* strains were manipulated by adding different concentrations of arabinose to the growth media.

### Generation and Complementation of the Deletion Mutants

The *brpR* gene was inactivated *in vitro* by deletion of the ORF of *brpR* (967-bp of 1335-bp) using the PCR-mediated linker-scanning mutation method as described previously (Kim et al., 2014). Briefly, pairs of primers BRPR01-F and -R (for amplification of the 5′ amplicon) or BRPR02-F and -R (for amplification of the 3′ amplicon) were designed and used (Supplementary Table S2). The resulting Δ*brpR* fragment was amplified by PCR using the mixture of both amplicons as the template and BRPR01-F and BRPR02-R as primers. Similar experimental procedures were adopted for amplification of the Δ*brpT* and Δ*brpS* fragments *in vitro*, except that primers BRPT01-F, BRPT01-R, BRPT02-F, and BRPT02-R (for 405-bp deleted *brpT*) and BRPS01-F, BRPS01-R, BRPS02-F, and BRPS02-R (for 627-bp deleted *brpS*) were used (Supplementary Table S2). The resulting Δ*brpR*, Δ*brpT*, and Δ*brpS* fragments were ligated into *Spe*I-*Sph*I-digested pDM4 (Milton et al., 1996) to generate pJN1302, pJN1607, and pSH1805, respectively (Supplementary Table S1). *E. coli* S17-1 λ*pir*, *tra* strain (Simon et al., 1983) containing pJN1302, pJN1607, or pSH1805 was used as a conjugal donor to JN111 to generate the *brpR* mutant JN131D, the *brpT* mutant JN161D, or the *brpS* mutant SH181D, respectively (Supplementary Table S1). Similarly, *E. coli* S17-1 λ*pir*, *tra* strain containing pJN1607 was used as a conjugal donor to JN131D to generate the *brpR brpT* double mutant JN162D (Supplementary Table S1). The conjugation and isolation of the transconjugants were conducted using the method described previously (Kim et al., 2011). In the same manner as above, the *brpT* mutant SH191 and the *brpS* mutant SH192 were generated from *V. vulnificus* strain ATCC 27562 using pJN1607 and pSH1805 (Supplementary Table S1).

To complement the *brpR* and *brpT* deletions, each ORF of *brpR* and *brpT* was amplified by PCR using a pair of specific primers, as listed in Supplementary Table S2. The amplified ORFs of *brpR* and *brpT* were cloned into pJK1113 (Lim et al., 2014) under an arabinose-inducible promoter P_BAD_ to create pJN1601 and pJN1602, respectively (Supplementary Table S1). The plasmids were transferred into the appropriate mutants by conjugation as described above.

### RNA Purification and Transcript Analysis

Total RNA was isolated by using an RNeasy^®^ mini kit (Qiagen, Valencia, CA, United States) from biofilms of the *V. vulnificus* strains grown on 24-well polystyrene microtiter plates (SPL, Seoul, South Korea) containing VFMG supplemented with 0.01% (w/v) arabinose for 24 h. For quantitative reverse transcription-PCR (qRT-PCR), the concentrations of total RNAs were measured by using a NanoDrop^TM^ One^C^ spectrophotometer (Thermo Scientific, Waltham, MA, United States), and cDNA was synthesized from 1 μg of the total RNA by using an iScript^TM^ cDNA synthesis kit (Bio-Rad, Hercules, CA, United States). Real-time PCR amplification of the cDNA was performed by using a CFX96 real-time PCR detection system (Bio-Rad) with pairs of specific primers (Supplementary Table S2), as described previously (Kim et al., 2012). Relative levels of the transcripts were calculated by using the glyceraldehyde-3-phosphate dehydrogenase (GAPDH) expression level as the internal reference for normalization (Williams et al., 2014).

For primer extension analysis, a 24-base primer CABAUP-R (Supplementary Table S2) complementary to the coding region of *cabA* was end-labeled with [γ-^32^P]-ATP and added to the RNA. The primer was then extended with SuperScript II reverse transcriptase (Invitrogen). The cDNA products were purified and resolved on a sequencing gel alongside sequencing ladders generated from pBH1402 with the same primer. The plasmid pBH1402 was constructed by cloning the 337-bp *cabA* upstream region extending from −230 to +107, amplified by PCR using a pair of primers CABAUP-F and -R (Supplementary Table S2), into pGEM-T Easy (Promega, Madison, WI, United States). The primer extension product was visualized using a phosphor image analyzer (BAS1500, Fujifilm, Tokyo, Japan).

### Construction of a Set of *cabA-luxCDABE* Transcriptional Fusions

The primer CABAR006 carrying a *Spe*I restriction site was used in conjunction with one of the primers carrying a *Sac*I restriction site, CABAR001, CABAR002. CABAR003, CABAR004, and CABAR005 (Supplementary Table S2), to amplify the DNA of *cabA* extending up to −212, −132, −106, −50, and +17 bp, respectively. The amplified DNA fragments were inserted into the *Spe*I-*Sac*I-digested pBBR-lux carrying promoterless *luxCDABE* genes (Lenz et al., 2004) to create five *cabA*-lux reporter constructs: pSH1704, pSH1705, pSH1706, pSH1707, and pSH1708 (Supplementary Table S1). The constructs were then transferred into the *V. vulnificus* strains by conjugation. The cellular luminescence of each culture grown to an *A*_600_ of 2.0 in LB medium supplemented with 0.1% arabinose was measured using a Tecan Infinite M200 microplate reader (Tecan, Männedorf, Switzerland) and expressed in arbitrary RLUs as described previously (Jeong et al., 2003).

### Protein Purification, EMSA, and DNase I Protection Assay

The ORFs of *brpT* and *brpS* were amplified by PCR using pairs of primers BRPT04-F and -R or BRPS04-F and -R (Supplementary Table S2) and subcloned into pET-28a(+) (Novagen, Madison, WI, United States), resulting in pSH1819 and pSH1823 (Supplementary Table S1), respectively. The His_6_-tagged BrpT and BrpS were expressed in *E. coli* BL21 (DE3) and purified by affinity chromatography using Ni-NTA agarose (Qiagen).

For EMSA, The 337-bp *cabA* upstream region, extending from −230 to +107, was amplified by PCR using unlabeled CABAUP-F and [γ-^32^P]-ATP-labeled CABAUP-R as primers (Supplementary Table S2). Similarly, the 388-bp *brpT* upstream region or 358-bp *brpS* upstream region were amplified by PCR using unlabeled BRPTUP-F or BRPSUP-F in conjunction with [γ-^32^P]ATP-labeled BRPTUP-R or BRPSUP-R as primers (Supplementary Table S2), respectively. For binding of BrpT, the labeled DNA (2.5 nM) was incubated with purified BrpT for 25 min at 30°C in a 20-μl reaction mixture containing 1 × BrpT-binding buffer (20 mM HEPES (pH 7.6), 1 mM EDTA, 10 mM (NH_4_)_2_SO_4_, 1 mM DTT, 0.2% (v/v) Tween 20, 30 mM KCl, 75 mM NaCl, 50 μM c-di-GMP) and 0.1 μg of poly(dI-dC) (Sigma-Aldrich, St. Louis, MO, United States). The protein-DNA binding reactions with BrpS were performed in the same manner as those with BrpT, except that 1 × BrpS-binding buffer (10 mM Tris–HCl (pH 7.9), 50 mM NaCl, 1 mM DTT, 0.1 mM EDTA, 50 μM c-di-GMP) was used. Electrophoretic analysis of the protein-DNA complexes was performed as described previously (Jang et al., 2017). When necessary, various concentrations of unlabeled DNA were added as competitors to the reaction mixture before incubation.

For DNase I protection assay, the same 337-bp *cabA* upstream region as above was amplified by PCR using unlabeled CABAUP-F and 6-carboxyfluorescein (6-FAM)-labeled CABAUP-R as primers (Supplementary Table S2). The binding of purified BrpT or BrpS to the labeled DNA (48 nM) and DNase I digestion of the protein-DNA complexes followed the procedures described previously (Lim and Choi, 2014), except that 1 × BrpT- or BrpS-binding buffer was used, respectively. The digested DNA products were precipitated with ethanol, eluted in H_2_O, and analyzed using an ABI 3730×l DNA analyzer (Applied Biosystems, Foster City, CA, United States) with Peak Scanner^TM^ Software v1.0 (Applied Biosystems).

### Quantitative and Structural Analyses of Biofilms

To quantify biofilms of the *V. vulnificus* strains, each well of the 96-well polystyrene microtiter plates (Nunc, Roskilde, Denmark) was inoculated with 200 μl of each culture diluted to an *A*_600_ of 0.05 in VFMG supplemented with 0.01% arabinose. After 24 h static incubation at 30°C, the biofilms were stained with 1% (w/v) crystal violet solution for 15 min and quantified by elution of crystal violet from the stained biofilms with ethanol and measurement of absorbance at 570 nm (*A*_570_) as described previously (Park et al., 2015a). To visualize the crystal violet-stained biofilms, biofilms of the strains were formed as described above but in a larger scale (1 ml) using 14 ml round-bottom test tubes (BD Biosciences, San Jose, CA, United States), and the stained biofilms were photographed using a digital camera (PowerShot G7X Mark II, Canon, Tokyo, Japan).

For structural analyses, biofilms of the strains were formed in flow cell chambers using the method described previously (Park et al., 2015a). Glass coverslips were attached on polycarbonate flow cells with individual channel dimensions of 1 × 4 × 40 mm. Each flow cell was inoculated with 100 μl of the culture diluted to *A*_600_ of 0.1, and inverted to allow bacteria to attach to the coverslip for 1 h without flow. Then VFMG supplemented with 0.01% arabinose was flowed at a constant rate of 8 ml/h using a Minipuls Evolution^TM^ peristaltic pump (Gilson, Villiers-le-Bel, France) to grow biofilms for 3 days.

For CLSM analysis, biofilms on the coverslips were stained with LIVE/DEAD *Bac*Light^TM^ bacterial viability kit containing SYTO 9 and propidium iodide (Invitrogen) for 15 min in the dark and visualized by CLSM (LSM710, Zeiss). The biofilm images were processed using Zeiss Zen software (Zeiss). For SEM analysis, biofilms on the coverslips were fixed and dehydrated as described previously (Park et al., 2015a). The resulting biofilms were mounted on an aluminum stub, coated with platinum using a putter coater (BAL-TEC SCD 005, BAL-TEC AG, Balzers, Liechtenstein), and visualized using SEM (Supra 55VP, Zeiss).

### Colony Morphology Assay

For analysis of colony morphology, 2 μl of each culture grown to *A*_600_ of 0.8 was spotted onto VFMG agar plate supplemented with 0.02% arabinose. The resulting colonies grown at 30°C for 36 h were visualized using a Stemi 305 stereomicroscope (Zeiss, Oberkochen, Germany) equipped with an Axiocam 105 color camera (Zeiss).

### Data Analysis

The mean and standard deviation (SD) values were calculated from at least three independent experiments. The experimental data were analyzed by Student’s *t*-test using GraphPad Prism 7.0 (GraphPad Software, San Diego, CA, United States). Significance of differences between experimental groups was accepted at a *p* value of <0.05.

## Data Availability Statement

All datasets generated for this study are included in the article/Supplementary Material.

## Author Contributions

All authors conceived and designed the research, analyzed and interpreted the data, reviewed the results, and approved the final version of the manuscript. S-HH, JP, and BL performed the experiments. S-HH and SC wrote the manuscript.

## Conflict of Interest

The authors declare that the research was conducted in the absence of any commercial or financial relationships that could be construed as a potential conflict of interest.
