# Supplementary material for: A Regulatory Network Controls cabABC Expression Leading to Biofilm and Rugose Colony Development in Vibrio vulnificus
Source: Front Microbiol. 2020 Jan 17;10:3063. doi: 10.3389/fmicb.2019.03063 (PMC6978666; doi:10.3389/fmicb.2019.03063)
Supplement: Supplementary file 1 [file Image_1.PDF]

Supplementary Figure S1

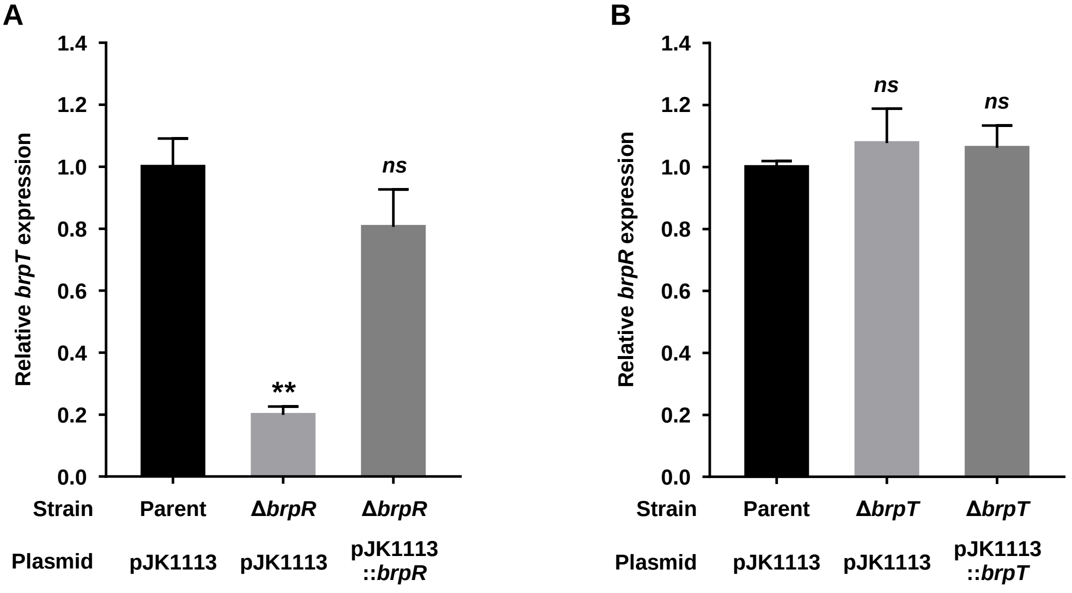

Figure S1. BrpR activates *brpT*, but BrpT does not affect *brpR*. Total RNA was isolated from biofilms of the *V. vulnificus* strains grown in microtiter plates. The level of *brpT* (A) or *brpR* (B) transcript was determined by qRT-PCR, and the parent strain was set to 1. Error bars represent the SD from three independent experiments. **, $p < 0.005$ relative to the parent strain; *ns*, not significant. Parent (pJK1113), parent strain; Δ*brpR* (pJK1113), *brpR* mutant; Δ*brpT* (pJK1113), *brpT* mutant; Δ*brpR* (pJK1113::*brpR*) and Δ*brpT* (pJK1113::*brpT*), complemented strains.
