# Supplementary material for: A Regulatory Network Controls cabABC Expression Leading to Biofilm and Rugose Colony Development in Vibrio vulnificus
Source: Front Microbiol. 2020 Jan 17;10:3063. doi: 10.3389/fmicb.2019.03063 (PMC6978666; doi:10.3389/fmicb.2019.03063)
Supplement: Supplementary file 2 [file Image_2.PDF]

Supplementary Figure S2

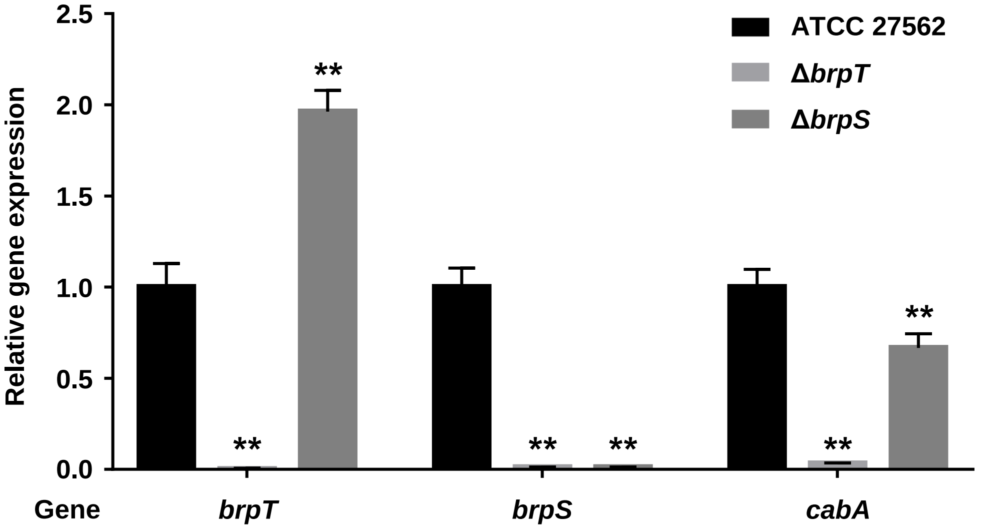

Figure S2. Effects of the *brpT* and *brpS* deletions on gene expression in strain ATCC 27562. Total RNA was isolated from biofilms of the *V. vulnificus* ATCC 27562 strains grown in microtiter plates. Intracellular c-di-GMP levels of the strains were elevated by introducing pJN1002 carrying *dcpA* under IPTG-inducible promoter and adding 1 mM IPTG to the media. The levels of *brpT*, *brpS*, and *cabA* transcripts were determined by qRT-PCR, and the parent strain was set to 1. Error bars represent the SD from three independent experiments. **, $p < 0.005$ relative to the parent strain. ATCC 27562, parent strain; Δ*brpT*, ATCC 27562 *brpT* mutant; Δ*brpS*, ATCC 27562 *brpS* mutant.
