# Supplementary material for: A Regulatory Network Controls cabABC Expression Leading to Biofilm and Rugose Colony Development in Vibrio vulnificus
Source: Front Microbiol. 2020 Jan 17;10:3063. doi: 10.3389/fmicb.2019.03063 (PMC6978666; doi:10.3389/fmicb.2019.03063)
Supplement: Supplementary file 3 [file Table_1.PDF]

Supplementary Table S1. Bacterial strains and plasmids used in this study

Strain or plasmid	Relevant characteristics[a]	Reference or source
Bacterial strains		
V. vulnificus		
JN111	CMCP6 with P_{BAD}-*dcpA*	(Park et al., 2015a)
JN131D	JN111 with Δ*brpR*	This study
JN161D	JN111 with Δ*brpT*	This study
JN162D	JN111 with Δ*brpR* Δ*brpT*	This study
SH181D	JN111 with Δ*brpS*	This study
ATCC27562	Clinical isolate	Laboratory collection
SH191	ATCC27562 with Δ*brpT*	This study
SH192	ATCC27562 with Δ*brpS*	This study
E. coli		
S17-1 λ*pir*	λ-*pir* lysogen; *thi pro hsdR* $hsdM^{+}$ *recA* RP4-2 Tc::Mu-Km::Tn7;Tp^{r} Sm^{r}; host for π-requiring plasmids	(Simon et al., 1983)
BL21 (DE3)	F^{-}, *ompT*, *hsdS* (r_B^{-}, m_B^{-}), *gal dcm* (DE3)	Laboratory collection
Plasmids		
pDM4	R6K γ *ori sacB*; suicide vector; *oriT* of RP4; Cm^{r}	(Milton et al., 1996)
pJN1302	pDM4 with Δ*brpR*; Cm^{r}	This study
pJN1607	pDM4 with Δ*brpT*; Cm^{r}	This study
pSH1805	pDM4 with Δ*brpS*; Cm^{r}	This study
pJK1113	pKS1101 with *nptI*; Ap^{r} Km^{r}	(Lim et al., 2014)
pJN1601	pJK1113 with *brpR*; Ap^{r} Km^{r}	This study
pJN1602	pJK1113 with *brpT*; Ap^{r} Km^{r}	This study
pET-28a(+)	His_6-tag fusion expression vector; Km^{r}	Novagen
pSH1819	pET-28a(+) with *brpT*; Km^{r}	This study
pSH1823	pET-28a(+) with *brpS*; Km^{r}	This study
pGEM-T Easy	PCR product cloning vector; Ap^{r}	Promega
pBH1402	pGEM-T Easy with 337-bp fragment of *cabA* upstream region; Ap^{r}	This study
pBBR-lux	Broad-host-range vector with *luxCDABE* operon; Cm^{r}	(Lenz et al., 2004)
pSH1704	pBBR-lux with 364-bp fragment of *cabA* upstream region; Cm^{r}	This study
pSH1705	pBBR-lux with 284-bp fragment of *cabA* upstream region; Cm^{r}	This study
pSH1706	pBBR-lux with 258-bp fragment of *cabA* upstream region; Cm^{r}	This study
pSH1707	pBBR-lux with 202-bp fragment of *cabA* upstream region; Cm^{r}	This study
pSH1708	pBBR-lux with 136-bp fragment of *cabA* upstream region; Cm^{r}	This study
pJN1002	pJH0311 with *dcpA*	(Park et al., 2015b)

[a] Tp^{r}, trimethoprim-resistant; Sm^{r}, streptomycin-resistant; Cm^{r}, chloramphenicol-resistant; Ap^{r}, ampicillin-resistant; Km^{r}, kanamycin-resistant.

References

Lenz, D.H., Mok, K.C., Lilley, B.N., Kulkarni, R.V., Wingreen, N.S., and Bassler, B.L. (2004). The small RNA chaperone Hfq and multiple small RNAs control quorum sensing in *Vibrio harveyi* and *Vibrio cholerae*. *Cell* 118(1), 69-82. doi: DOI 10.1016/j.cell.2004.06.009.

Lim, J.G., Bang, Y.J., and Choi, S.H. (2014). Characterization of the *Vibrio vulnificus* 1-Cys peroxiredoxin Prx3 and regulation of its expression by the Fe-S cluster regulator IscR in response to oxidative stress and iron starvation. *J Biol Chem* 289(52), 36263-36274. doi: 10.1074/jbc.M114.611020.

Milton, D.L., O'Toole, R., Horstedt, P., and Wolf-Watz, H. (1996). Flagellin A is essential for the virulence of *Vibrio anguillarum*. *J Bacteriol* 178(5), 1310-1319. doi: 10.1128/jb.178.5.1310-1319.1996.

Park, J.H., Jo, Y., Jang, S.Y., Kwon, H., Irie, Y., Parsek, M.R., et al. (2015a). The *cabABC* Operon Essential for Biofilm and Rugose Colony Development in *Vibrio vulnificus*. *PLoS Pathog* 11(9), e1005192. doi: 10.1371/journal.ppat.1005192.

Park, J.H., Lim, J.G., and Choi, S.H. (2015b). Effects of elevated intracellular cyclic di-GMP levels on biofilm formation and transcription profiles of *Vibrio vulnificus*. *Food Science and Biotechnology* 24(2), 771-776. doi: 10.1007/s10068-015-0100-5.

Simon, R., Priefer, U., and Puhler, A. (1983). A Broad Host Range Mobilization System for Invivo Genetic-Engineering - Transposon Mutagenesis in Gram-Negative Bacteria. *Bio-Technology* 1(9), 784-791. doi: DOI 10.1038/nbt1183-784.
