# Supplementary material for: A Regulatory Network Controls cabABC Expression Leading to Biofilm and Rugose Colony Development in Vibrio vulnificus
Source: Front Microbiol. 2020 Jan 17;10:3063. doi: 10.3389/fmicb.2019.03063 (PMC6978666; doi:10.3389/fmicb.2019.03063)
Supplement: Supplementary file 4 [file Table_2.PDF]

**Supplementary Table S2.** Oligonucleotides used in this study

| Oligonucleotide | Oligonucleotide sequence (5' →3')[a] | Use |
|---|---|---|
| For mutant construction | | |
| BRPR01-F | TCAGCGCCGATGCTGATAACC | Deletion of *brpR* ORF |
| BRPR01-R | AGGGATCCCGATAGTAAACGCAG | |
| BRPR02-F | CGGGATCCCCATCATGGCTAAGG | Deletion of *brpR* ORF |
| BRPR02-R | GTCGGGTGATGACGAAGCCATG | |
| BRPT01-F | TGTATTTGGCGTTCTTTGAC | Deletion of *brpT* ORF |
| BRPT01-R | TATCGGATCCATCCAGTTTCAGCCA | |
| BRPT02-F | CCATGGATCCGATAAGGCAGGTG | Deletion of *brpT* ORF |
| BRPT02-R | CACCTTGGCCACCGATCAAA | |
| BRPS01-F | AAGAGCTGCGTGAGTTGTC | Deletion of *brpS* ORF |
| BRPS01-R | AAGATGTGGGCTTGTTTTGACTCTCCATGC | |
| BRPS02-F | TCAAAACAAGCCCACATCTTCTACGATC | Deletion of *brpS* ORF |
| BRPS02-R | TTCCGGCTCTAAGTCTTC | |
| For mutant complementation | | |
| BRPR03-F | GCCCATGGCTACTCAGTTTAAGATG | Amplification of *brpR* ORF |
| BRPR03-R | TAGTCGACGCTTTGGGTGATTTAAAC | |
| BRPT03-F | GACCATGGCAGACACAACGATTG | Amplification of *brpT* ORF |
| BRPT03-R | GAGCATGCCCTATAGAAATAGTGGC | |
| For protein overexpression | | |
| BRPT04-F | GTTTAACTTTAAGAAGGAGATATACCATGG CAGACACAACGATTG | Amplification of *brpT* ORF |
| BRPT04-R | CAGTGGTGGTGGTGGTGGTGTAAATTCCGT TTTGCCCACG | |
| BRPS04-F | GTTTAACTTTAAGAAGGAGATATACCATGG AGAGTCAAAACAAGGAAG | Amplification of *brpS* ORF |
| BRPS04-R | CAGTGGTGGTGGTGGTGGTGGATCGTAGA AGATGTGGGAG | |
| For qRT-PCR | | |
| CABA-qRT-F | TTGGTTGCTGGCTCTGGTGAC | Quantification of *cabA* expression |
| CABA-qRT-R | ACTGTCTATACGCACTGTGTCCTC | |
| BRPR-qRT-F | TTAGCGATGCCATTGAGACTGAAC | Quantification of *brpR* expression |
| BRPR-qRT-R | GGTTGGTGGTACTTATGAGCCTTG | |
| BRPT-qRT-F | GAAGCTGTGTCGCGGGATTG | Quantification of *brpT* expression |
| BRPT-qRT-R | TGTGGCTCTTCCTTCTTCGCTC | |
| BRPS-qRT-F | TATGGTCGTTCATTTGCTCACTCC | Quantification of *brpS* expression |
| BRPS-qRT-R | AATCAGCACCTCTTGAACTTCTG | |
| BRPA-qRT-F | CATCGGCTTTATGGCCTTGC | Quantification of *brpA* expression |
| BRPA-qRT-R | GCGCTTTCGGCAAAGAGAAT | |
| GAPDH-qRT-F | TGAAGGCGGTAACCTAATCG | Quantification of *gapdh* expression |
| GAPDH-qRT-R | TACGTCAACACCGATTGCAT | |
| For primer extension analysis, EMSA, or DNase I protection assay | | |
| CABAUP-F | CGTGGGCAAAACGGAATTTATAAAA | Amplification of *cabA* upstream region and extension of *cabA* transcript |
| CABAUP-R | CATCTGCAGTTCCAGAATAAACAGCC | |
| BRPTUP-F | CAAATCAGCAAATTGCACGCAAGC | Amplification of *brpT* upstream region |
| BRPTUP-R | CGTTGTGTCTGCCATATTACAACCC | |
| BRPSUP-F | AAGAGGCCAGTCAAGAGCCG | Amplification of *brpS* upstream region |
| BRPSUP-R | CCTTGTTTTGACTCTCCATGCTCCC | |
| For promoter deletion assay | | |

| | | |
|---|---|---|
| CABAR001 | ATGAGCTCTATAAAACCGCCTGGC | Deletion of *cabA* regulatory region |
| CABAR002 | ATGAGCTCGCAATAACTGTTTGTTTTATC | |
| CABAR003 | ATGAGCTCTAAGCGTTGAAAAGTATGAG | |
| CABAR004 | ATGAGCTCCAACCGAAAATCAAACTC | |
| CABAR005 | ATGAGCTCGTCAGATGTTGTTTAACG | |
| CABAR006 | CTACTAGTCTACGAACTTGTCATCACTAC | |

[a] Regions of oligonucleotides not complementary to the corresponding genes are underlined.
